# Association between dietary carotenoid intakes and the risk of heart failure in males and females: a cross-sectional study of NHANES, 2009–2018

**DOI:** 10.3389/fgwh.2025.1568812

**Published:** 2025-05-27

**Authors:** Juan Gao, Yan-You Xie, Yi-Chen Zang, Kai Tan, Pei-Hui Li, Hai-Yang Yu, Zhe-Xun Lian, Jian-Xun Wang

**Affiliations:** ^1^ Department of Cardiology, The Affiliated Hospital of Qingdao University, Qingdao University, Qingdao, China; ^2^ School of Basic Medicine, Qingdao University, Qingdao, China; ^3^ Department of Abdominal Ultrasound, The Affiliated Hospital of Qingdao University, Qingdao, China; ^4^ Department of Radiology, The Affiliated Hospital of Qingdao University, Qingdao, China

**Keywords:** carotenoid intakes, heart failure, gender difference, NHANES, cross-sectional study

## Abstract

**Purpose:**

Heart failure (HF) is a major contributor to morbidity and mortality among males and females worldwide. However, the difference in predisposition, progression, and management of HF between males and females remains underexplored. This study aimed to investigate the association between dietary carotenoid intake and HF using data from a nationally representative sample of adults in the US.

**Patients and methods:**

The National Health and Nutrition Examination Survey was conducted from 2009 to 2018. A total of 22,119 participants (10,519 males and 11,600 females) aged 20–80 years were included in this study. Logistic regression analyses and smooth curve fitting were used to explore the association between carotenoid intake and the risk of HF in males and females.

**Results:**

The odd ratios with 95% confidence intervals of dietary carotenoid intake for individuals with current HF, after adjusting for confounders in the model were 0.34 (0.13, 0.85; *P* for trend = 0.016) in females and 1.35 (0.74, 2.44; *P* for trend = 0.255) in males, comparing the highest to the lowest quartile. Smooth curve fitting suggested that total carotenoid intake was negatively associated with the risk of HF in females. The sex-based difference in this association was statistically significant.

**Conclusions:**

Higher dietary carotenoid intake was associated with lower odds of having current HF in US females but not in males. However, this was a cross-sectional study, no causal relationship could be drawn, and the results should be interpreted with caution.

## 1 Introduction

Heart failure (HF) is a clinical syndrome caused by structural and functional defects in the myocardium, resulting in impaired ventricular filling or ejection (1, 2). HF affects more than 26 million people worldwide and is a leading cause of hospitalization in Europe and the USA (3, 4). With environmental and lifestyle changes, the incidence of HF has increased over the past few decades, with an additional 100 million HF cases expected by 2025 (4). Although considerable progress has been made in studying sex-based differences in HF, much remains unclear. HF affects males and females equally; however, important sex-based differences in the impact of traditional risk factors, such as hypertension, diabetes mellitus, tobacco consumption, and obesity, predispose females to HF to a greater extent than, males (5, 6). In addition, the presentation of HF varies between sexes, with females presenting with more severe symptoms and manifesting HF syndrome in the backdrop of a higher left ventricular ejection fraction (7, 8). Therefore, broader inclusion of females in clinical and basic science studies is urgently required to better understand the pathobiological and clinical sex-based differences that underlie HF.

Increasing evidence has shown that metabolic factors, such as high-fat and high-salt diets, are closely related to the occurrence and development of HF. Epidemiological evidence suggests that a high intake of saturated fats and simple sugars is associated with an increased incidence of HF. In addition, a greater intake of eggs or high-fat dairy foods and a lower intake of whole grains were each associated with an increased risk of HF in a large biracial cohort (9, 10). In contrast, adherence to Mediterranean diets and DASH diets, which are characterized by an intake of plant-based foods (mainly vegetables and fruits), revealed a protective effect on the incidence of HF or worsening of cardiac function parameters. These benefits were signiﬁcantly greater compared to patients who did not adhere to these dietary patterns (11–13).

Carotenoids, widely found in fruits, vegetables, and seaweed species, have anti-inflammatory and antioxidative effects and have been shown to be related to hypertension, non-alcoholic fatty liver disease, cancer, and other chronic diseases (14–16). However, evidence regarding the association between carotenoid intake and HF remains lacking, especially in males and females. Moreover, the associations among age, body mass index (BMI), and other individual characteristics have not been well described or analyzed. Therefore, this study aimed to explore the relationship between dietary carotenoid intake and HF using data from the National Health and Nutrition Examination Survey (NHANES) (2009–2018), a dataset that provides a nationally representative sample of the US population.

## 2 Material and methods

### 2.1 Data source

This study used data from NHANES, a nationally representative cross-sectional survey designed to assess the health and nutrition of the U.S. population. The research protocols were approved by the National Center for Health Statistics Ethics Committee, and all participants provided written informed consent.

Data were gathered from five NHANES cycles spanning 2009 to 2018, comprising 49,693 participants. After excluding 6,645 individuals with missing carotenoid intake data and 20,929 with incomplete HF information, a total of 22,119 participants remained for the final analysis.

### 2.2 Heart failure

HF in the present study was defined using self-report or proxy-report data from the NHANES Medical Conditions section (variable prefix MCQ), which includes personal interview data on a broad range of health conditions and medical history for both children and adults. Trained interviewers asked the participants the following question: “Has a doctor or other health professional ever told you that you had congestive heart failure?”. A response of “yes” was classified as having HF.

### 2.3 Dietary carotenoid intake

Two 24-h dietary recall interviews were performed to obtain dietary carotenoid intake (*μ*g/day) by trained interviewers, with the first conducted in-person at the Mobile Examination Center and the second by phone 3 to 10 days later. These interviews provided detailed information on foods and beverages (including water) consumed in the previous 24 h, allowing for the assessment of energy, nutrients, and other dietary element intakes. In this study, the intake of α-carotene, β-carotene, β-cryptoxanthin, lycopene, and lutein with zeaxanthin was averaged across both recalls, with total carotenoids defined as the sum of these five.

### 2.4 Other covariates

Based on previous studies, we selected covariates as potential confounding factors and included them in the adjusted model. The included variables were categorized as follows: demographic factors such as age, ethnicity, and poverty income ratio (PIR), which represents household income; lifestyle-related factors, including smoking, alcohol consumption, physical activity, sodium intake and energy intake; anthropometric measures, such as BMI; and clinical or biochemical indicators, such as total cholesterol, hypertension, diabetes, and coronary heart disease. Additionally, the use of antihypertensive drugs was considered.

BMI was calculated as weight divided by height squared (kg/m^2^). Blood pressure was measured three consecutive times, and the average value was calculated after the participants rested in a seated position for 5 min. Smoking status was categorized as “current smoker” or “non-smoker” based on the question, “Do you now smoke cigarettes?”. Alcohol consumption status was assessed by asking participants, “Have you consumed at least 12 alcohol drinks/1 yr?”. MET-minutes/week ≥600 was classified as physically active. Diabetes was defined according to the American Diabetes Association criteria, which included self-reported diabetes, use of glucose-lowering medications, fasting blood glucose ≥ 126 mg/dl, 2-h OGTT glucose ≥ 200 mg/dl, or HbA1c ≥ 6.5%. Hypertension was defined by one or more of the following: self-reported hypertension, use of antihypertensive medications, an average diastolic blood pressure ≥ 90 mmHg, or an average systolic blood pressure ≥ 140 mmHg.

### 2.5 Statistical analysis

Baseline characteristics are summarized as means ± SD for continuous variables and frequencies (%) for categorical variables. Subsequently, these variables were compared across quartiles of dietary carotenoid intake using ANOVA and chi-square tests. Given the skewed distribution of dietary carotenoid intake, data were log-transformed (Lg) for statistical analysis. Carotenoids intake was adjusted for total energy intake using the residual method. Additionally, due to sex-specific differences in the relationship between dietary carotenoid intake and HF, all analyses were stratified according to sex. Logistic regression models were used to assess the independent association between dietary carotenoid intake and HF, with results reported as odds ratios (OR) and 95% confidence intervals (CI). Three models were applied: Model 1 was not adjusted for any variables; Model 2 was adjusted for age, race, BMI, poverty income ratio, smoking history, alcohol consumption, total cholesterol, hypertension, diabetes, coronary heart disease, antihypertensive drugs, physical activity, sodium intake. The trend was tested by converting the dietary carotenoid intake quartile categories into continuous data. A smoothed curve was fitted to assess the dose-response relationship between dietary carotenoid intake and HF, with covariate adjustments. Stratified analyses and interaction tests were conducted based on age (<\65 or ≥65 years), BMI (<25 or ≥25 kg/m^2^), current smoker (yes or no), alcohol drinker (yes or no), total cholesterol (<5.2 or ≥5.2 mmol/L), hypertension (yes or no), diabetes (yes or no).

Data analysis was conducted using Empower software and R 4.0.3, with the significance level set at *P* < 0.05.

## 3 Results

### 3.1 Participants' characteristics

**Figure 1:**
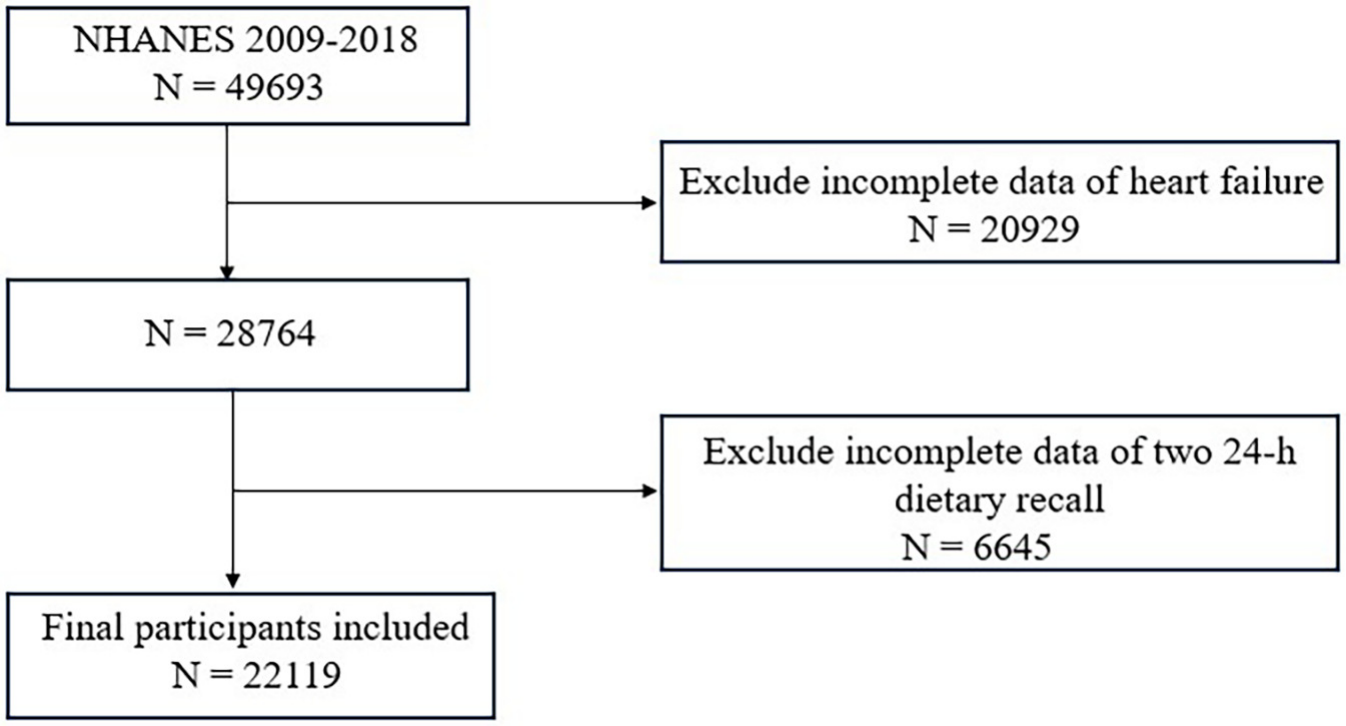
Flowchart of the sample selection from NHANES 2009–2018.

A total of 22,119 participants were included in this study. A flowchart of the study cohort selection is shown in Figure 1. The characteristics of the study population are shown in Table 1 (male) and Table 2 (female). Among males, participants with a higher total dietary carotenoid intake were more likely to be non-Hispanic white and were more likely to be rich. They also had a higher total energy intake but higher HDL-C levels. Additionally, they have a lower prevalence of smoking and hypertension. The prevalence of HF was lower among those with lower total dietary carotenoid intake. In females, these aspects were significantly different from males, and we found that participants with a higher dietary total carotenoid intake had a higher drinking prevalence and lower prevalence of hypertension and diabetes. Most importantly, the prevalence of HF was lower in those with lower total dietary carotenoid intake.

**Table 1:** Baseline characteristics of study participants stratified by quintiles of dietary carotenoid intakes among males.

Characteristics	Dietary carotenoids intake(µg/day)				*P*-value
	Quartile 1	Quartile 1	Quartile 1	Quartile 1	
	(<3,293.0)	(3,293.0–6,737.0)	(6,737.0–13,006.0)	(≥ 13,006.0)	
Males, *N*	2,630	2,629	2,630	2,630	
Age, years	51.15 ± 18.19	49.85 ± 17.74	49.99 ± 17.50	49.60 ± 17.16	0.007
Ethnicity					<0.001
Non-Hispanic White, *N* (%)	1,048 (39.85%)	1,105 (42.03%)	1,140 (43.35%)	1,171 (44.52%)	
Non-Hispanic Black, *N* (%)	752 (28.59%)	546 (20.77%)	460 (17.49%)	508 (19.32%)	
Mexican American, *N* (%)	308 (11.71%)	404 (15.37%)	398 (15.13%)	334 (12.70%)	
Other Hispanic, N(%)	256 (9.73%)	243 (9.24%)	263 (10.00%)	216 (8.21%)	
Other races, N(%)	266 (10.11%)	331 (12.59%)	369 (14.03%)	401 (15.25%)	
BMI, kg/m2	29.02 ± 6.39	29.12 ± 6.16	28.78 ± 6.22	28.87 ± 6.06	0.205
Poverty income ratio	2.33 ± 1.54	2.50 ± 1.61	2.75 ± 1.65	2.88 ± 1.67	<0.001
Current smoker, N(%)	751 (49.02%)	616 (42.96%)	517 (38.55%)	474 (37.83%)	<0.001
Alcohol drinker, N(%)	1,783 (75.33%)	1,853 (77.40%)	1,830 (77.02%)	1,877 (77.56%)	0.239
Total cholesterol, mmol/L	4.82 ± 1.10	4.88 ± 1.12	4.90 ± 1.10	4.88 ± 1.05	0.045
Triglyceride, mmol/L	1.45 ± 1.38	1.55 ± 1.47	1.53 ± 1.44	1.48 ± 1.26	0.335
HDL-C, mmol/L	1.23 ± 0.37	1.24 ± 0.38	1.24 ± 0.35	1.27 ± 0.38	0.008
LDL-C, mmol/L	2.90 ± 0.92	2.93 ± 0.95	2.93 ± 0.90	2.89 ± 0.93	0.555
Diabetes, *N* (%)	399 (15.17%)	391 (14.87%)	383 (14.57%)	376 (14.30%)	0.828
Hypertension, *N* (%)	1,068 (40.64%)	998 (38.08%)	921 (35.05%)	925 (35.20%)	<0.001
Heart failure, *N* (%)	122 (4.64%)	93 (3.54%)	93 (3.54%)	88 (3.35%)	0.056
Total energy intake, kcal	1,991.00 ± 786.72	2,277.94 ± 796.77	2,392.18 ± 827.66	2,655.14 ± 1,010.19	<0.001

BMI, body mass index; HDL-C, high-density lipoprotein cholesterol; LDL-C, low-density.

lipoprotein cholesterol.

Data are presented as number (%) or mean ± standard deviation.

**Table 2:** Baseline characteristics of study participants stratified by quintiles of dietary carotenoid intakes among females.

Characteristics	Dietary carotenoids intake(µg/day)				*P*-value
	Quartile 1	Quartile 1	Quartile 1	Quartile 1	
	(<2,910.5)	(2,910.5–6,092.0)	(6,092.0–11,668.5)	(≥ 11,668.5)	
Females, *N*	2,900	2,900	2,899	2,901	
Age, years	49.86 ± 17.76	49.67 ± 17.73	49.26 ± 17.34	49.74 ± 17.04	0.58
Ethnicity					<0.001
Non-Hispanic White, *N* (%)	1,162 (40.07%)	1,189 (41.00%)	1,208 (41.67%)	1,181 (40.71%)	
Non-Hispanic Black, *N* (%)	834 (28.76%)	629 (21.69%)	536 (18.49%)	578 (19.92%)	
Mexican American, *N* (%)	333 (11.48%)	431 (14.86%)	498 (17.18%)	381 (13.13%)	
Other Hispanic, *N* (%)	334 (11.52%)	313 (10.79%)	291 (10.04%)	305 (10.51%)	
Other races, *N* (%)	237 (8.17%)	338 (11.66%)	366 (12.63%)	456 (15.72%)	
BMI, kg/m^2^	30.67 ± 8.07	30.27 ± 8.03	29.75 ± 7.57	29.20 ± 7.55	<0.001
Poverty income ratio	2.06 ± 1.50	2.41 ± 1.60	2.58 ± 1.64	2.72 ± 1.68	<0.001
Current smoker, *N* (%)	666 (56.83%)	487 (47.79%)	391 (42.45%)	325 (34.43%)	<0.001
Alcohol drinker, *N* (%)	1,350 (54.06%)	1,411 (55.66%)	1,435 (55.62%)	1,479 (58.00%)	0.044
Total cholesterol, mmol/L	5.00 ± 1.06	5.02 ± 1.13	5.08 ± 1.05	5.08 ± 1.01	0.009
Triglyceride, mmol/L	1.31 ± 0.81	1.33 ± 1.55	1.26 ± 0.92	1.20 ± 0.85	0.009
HDL-C, mmol/L	1.43 ± 0.41	1.48 ± 0.42	1.51 ± 0.43	1.53 ± 0.42	<0.001
LDL-C, mmol/L	2.92 ± 0.91	2.90 ± 0.92	2.99 ± 0.94	2.93 ± 0.89	0.059
Diabetes, N (%)	422 (14.56%)	391 (13.50%)	344 (11.88%)	289 (9.97%)	<0.001
Hypertension, *N* (%)	1,186 (40.95%)	1,093 (37.74%)	1,000 (34.52%)	1,033 (35.62%)	<0.001
Heart failure, *N* (%)	108 (3.72%)	94 (3.24%)	71 (2.45%)	49 (1.69%)	<0.001
Total energy intake, kcal	1,514.09 ± 581.04	1,700.20 ± 580.34	1,815.02 ± 607.70	1,960.01 ± 662.73	<0.001

BMI, body mass index; HDL-C, high-density lipoprotein cholesterol; LDL-C, low-density.

lipoprotein cholesterol.

Data are presented as number (%) or mean ± standard deviation.

### 3.2 Association between dietary carotenoid intakes and the risk of heart failure

**Figure 2:**
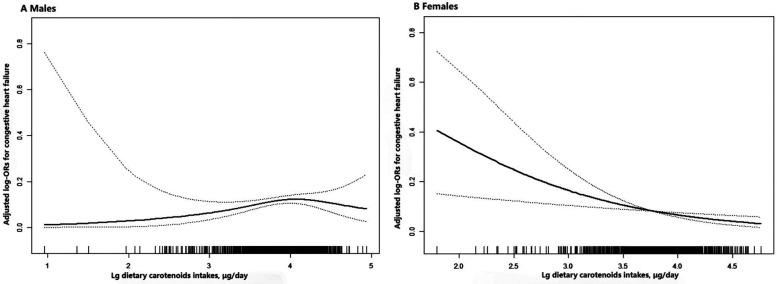
Association between dietary carotenoid intakes and the risk of HF by sex. **(A)** males **(B)** females. The solid line and dashed line represent the estimated values and their corresponding 95% confidence interval, respectively. Adjustment factors included age, race, BMI, poverty income ratio, smoking history, alcohol consumption, total cholesterol, hypertension, diabetes, coronary heart disease, antihypertensive drugs, physical activity, sodium intake.

The ORs with 95% CIs for HF across quartiles of dietary carotenoids and their total values are shown in Table 3. Compared to the Q1 reference group, the odds of developing HF in females revealed a gradual decrease across the Q2 (OR: 0.89; 95% CI: 0.44, 1.79), Q3 (OR: 0.62; 95% CI: 0.27, 1.40), and Q4 (OR: 0.34; 95% CI: 0.13, 0.85) groups (*P* for trend = 0.016), after fully adjusting for confounding variables. A one-unit increase in Lg carotenoid intake was associated with a 66% lower risk of HF (OR: 0.34; 95% CI: 0.17, 0.68; *P* = 0.003). In contrast, for males, dietary carotenoid intake in Model 3, after full adjustment, was not associated with the risk of HF, regardless of whether the intake was treated as a continuous (OR: 1.46; 95%CI: 0.85, 2.48; *P* = 0.167) or categorical variable (*P* for trend = 0.255). Specifically, compared to the Q1 reference group, the odds of developing HF in males showed no significant trend across the Q2 (OR: 1.20; 95% CI: 0.67, 2.14), Q3 (OR: 1.46; 95% CI: 0.81, 2.63), and Q4 (OR: 1.35; 95% CI: 0.74, 2.44) groups. These results are consistent with the findings shown by the smoothed curves (Figure 2).

**Table 3:** Association of dietary carotenoids intakes with the risk of HF among males and females.

Lg carotenoids intake^a^, µg	Evens (%)	CHF OR (95%CI), *P*-value		
		Model 1	Model 2	Model 3
Males				
Continuous	396 (3.76%)	1.16 (0.90, 1.49) 0.255	1.20 (0.89, 1.60) 0.228	1.46 (0.85, 2.48) 0.167
Quartile				
Q1 (<3,293.0)	122 (4.64%)	Reference	Reference	Reference
Q2 (≥3,293.0 to <6,737.0)	93 (3.54%)	1.44 (1.08, 1.92) 0.014	1.26 (0.91, 1.73) 0.162	1.20 (0.67, 2.14) 0.534
Q3 (≥6,737.0 to <13,006.0)	93 (3.54%)	1.19 (0.88, 1.61) 0.252	1.10 (0.79, 1.54) 0.554	1.46 (0.81, 2.63) 0.204
Q4 (≥13,006.0)	88 (3.35%)	1.30 (0.96, 1.74) 0.086	1.24 (0.90, 1.72) 0.195	1.35 (0.74, 2.44) 0.326
*P* for trend		0.253	0.355	0.255
Females				
Continuous	322 (2.78%)	0.67 (0.53, 0.86) 0.002	0.70 (0.52, 0.94) 0.017	0.34 (0.17, 0.68) 0.003
Quartile				
Q1 (<2,910.5)	108 (3.72%)	Reference	Reference	Reference
Q2 (≥2,910.5 to <6,092.0)	94 (3.24%)	1.10 (0.83, 1.47) 0.508	0.97 (0.70, 1.33) 0.828	0.89 (0.44, 1.79) 0.74
Q3 (≥6,092.0 to <11,668.5)	71 (2.45%)	0.81 (0.59, 1.10) 0.180	0.80 (0.57, 1.13) 0.202	0.62 (0.27, 1.40) 0.251
Q4 (≥11,668.5)	49 (1.69%)	0.62 (0.44, 0.87) 0.005	0.66 (0.46, 0.94) 0.023	0.34 (0.13, 0.85) 0.022
*P* for trend		0.001	0.013	0.016
*P* value for interaction*		0.003	0.009	0.002

Model 1 was adjusted for none.

Model 2 was adjusted for age, race, BMI, poverty income ratio.

Model 3 was adjusted for age, race, BMI, poverty income ratio, smoking history, alcohol consumption, total cholesterol, hypertension, diabetes, coronary heart disease, antihypertensive drugs, physical activity, sodium intake.

^a^ Carotenoids intake was adjusted for total energy intake using the residual method.

* *P* value for interaction test: 2-way interaction of sex and dietary carotenoids intakes (continuous) on CHF.

### 3.3 The difference between males and females

Subsequently, we compared the differences between males and females. In Model 1, without adjustment, a significant difference was observed between males and females (*P* = 0.003). After adjusting for age, race, BMI, and poverty income ratio in Model 2 (*P* = 0.009) and for all confounding factors in Model 3 (*P* = 0.002), the results remained stable and statistically significant.

### 3.4 Subgroup analyses

**Figure 3:**
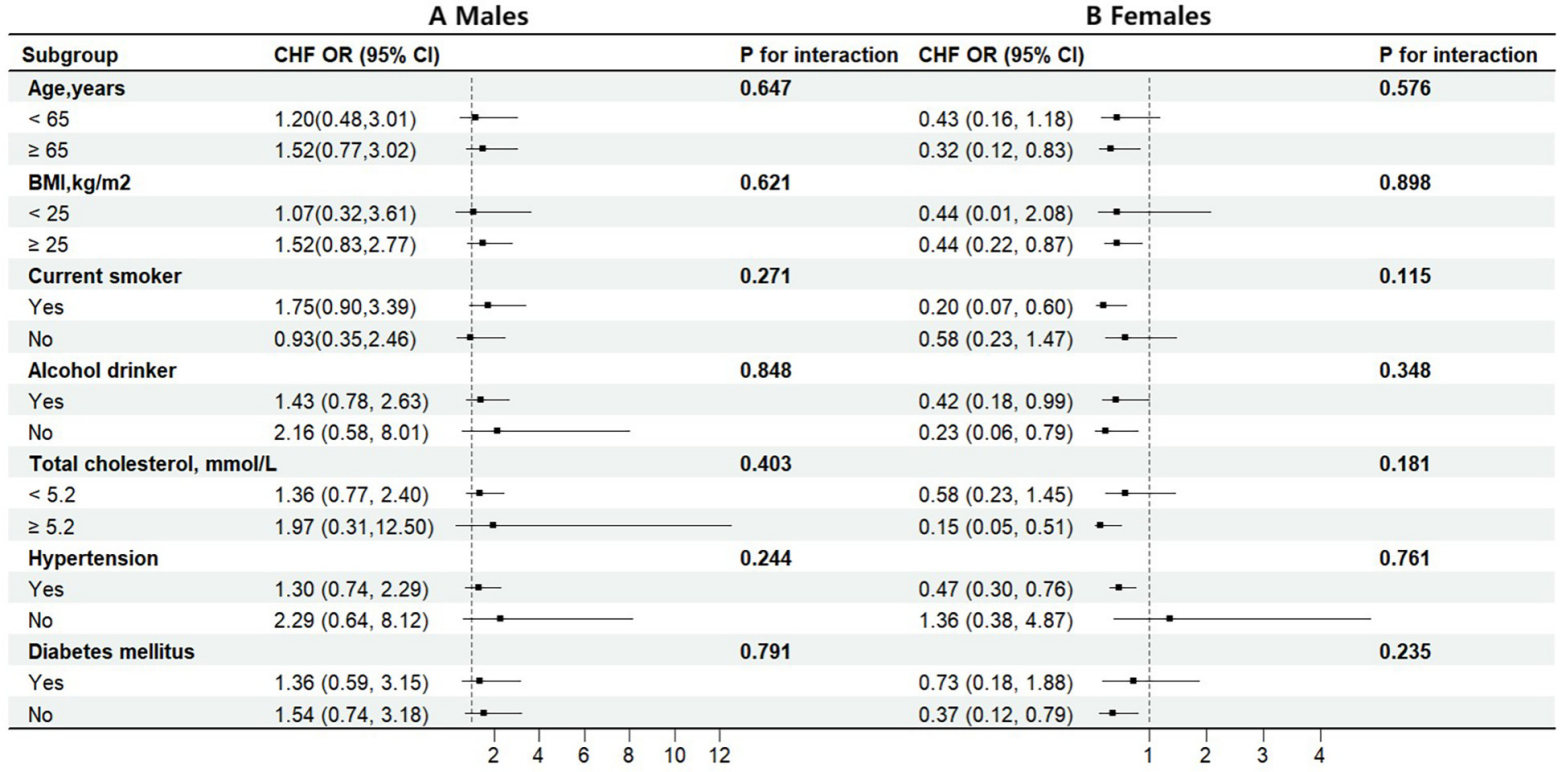
Stratified analyses by potential modifiers of the association between dietary carotenoids intakes and the risk of HF by sex **(A)** males **(B)** females. Each black square represents the effect size of the study, while the line indicates the 95% confidence interval CI. Each subgroup analysis adjusted for age, race, BMI, poverty income ratio, smoking history, alcohol consumption, total cholesterol, hypertension, diabetes, coronary heart disease, antihypertensive drugs, physical activity, sodium intake, except for the stratifying variable.

Additionally, stratified analyses were conducted to examine the association between dietary carotenoid intake and the risk of HF in different subgroups according to sex. Each subgroup analysis was adjusted for age, race, BMI, poverty income ratio, smoking, alcohol consumption, total cholesterol, hypertension, diabetes, coronary heart disease, antihypertensive drugs, and energy intake except for the stratifying variable. The interaction test for carotenoid intake with stratifying variables yielded a *P* value greater than 0.05, suggesting that the relationship between carotenoids and the risk of HF was stable in different subgroups (Figure 3).

## 4 Discussion

In this cross-sectional analysis, we used data from NHANES (2009–2018) to investigate the relationship between dietary carotenoid intake and the risk of HF. To our knowledge, this is the first study to examine the association between carotenoid intake and risk of HF, focusing on sex differences. Carotenoid intake was found to be associated with lower odds of HF in females but not in males. After adjusting for age, race, BMI, and other potential confounding factors, these relationships remained significant. In the stratified analysis, a stable association was found between carotenoid intake and the risk of HF across sex.

HF is a major healthcare issue given its high prevalence, incidence, rate of comorbidities, related high healthcare costs, and poor outcomes. HF affects approximately 25 million people worldwide and is a major public health problem (17, 18). The estimated prevalence varies between 1% and 3%; however, a 46% increase is projected by 2030 (19). Major pathogenic mechanisms leading to HF include increased hemodynamic overload, ischemia-related dysfunction, ventricular remodeling, and excessive neurohumoral stimulation (20, 21). Increased stress and reactive oxygen species (ROS) levels are the main reasons for increased HF. At the same time, HF was associated with increased oxidative stress and inflammation. Oxidative stress caused by an imbalance between prooxidants and antioxidants can be given as one of the causes of HF (22–24).

Carotenoids are fat-soluble, highly unsaturated pigments naturally present in plants, fungi, algae, and photosynthetic bacteria. They act as antioxidant, protecting against oxidative damage, and reducing inflammation by inhibiting the production of pro-inflammatory cytokines (25). Animal and human studies have found that carotenoids can significantly reduce obesity and fatty liver, lower blood sugar, improve liver fibrosis in cirrhosis, and reduce the risk of cardiovascular disease and erythema formation while lowering glycated hemoglobin and fasting plasma glucose levels (26, 27).

We speculate that carotenoids may be used to change the course of HF owing to their highly effective oxygen-quenching and ROS-scavenging properties (28). Carotenoids act as antioxidants that protect against oxidative damage and reduce inflammation by inhibiting the production of proinflammatory cytokines. By mitigating these processes, carotenoids may contribute to HF in patients (29, 30). Dietary patterns may also be of particular importance, as evidenced by the REGARDS trial that found a 72% higher risk of HF in those following a “Southern dietary pattern,” a common practice among Black females (31). Research also showed that high-fiber diet positively regulates the composition of gut microbiota, nutritional status and microinflammatory level in chronic HF patients, thereby improving patients' quality of life (32). However, due to the scarcity of research on the relationship between carotenoid intake and HF, we cannot directly establish a direct causal link between carotenoids and HF. On the other hand, whether HF patients will undergo changes in their dietary patterns after diagnosis is also a question worthy of our attention. We anticipate further research outcomes in the future to validate this relationship, thereby offering more prevention methods and treatment opportunities for HF patients.

Sex differences in traditional risk factors as well as sex-speciﬁc risk factors inﬂuence the prevalence and manifestation of HF in unique ways (33). Inherent biological differences in the structure and function of the cardiovascular system between males and females underlie sex-based differences in risk factors, pathophysiology, clinical presentation, diagnosis, and response to treatment (34, 35). Diabetes mellitus is independently associated with the risk of HF, however, to a greater extent in females than in males. Data from the Framingham Heart Study found a 2-fold greater risk in males but a 5-fold greater risk of HF in females with diabetes mellitus (7, 36). Hypertension also portends a greater risk of HF in females than males, with an estimated 3-fold vs. 2-fold increased risk of HF among females and males with hypertension compared to without, respectively (37, 38).

Obesity is a well-established risk factor for incident HF and is independently associated with unfavorable structural and hemodynamic cardiovascular changes (39). The underlying mechanisms are thought to be related to increased blood volume coupled with limited ventricular distensibility, mediated, at least in part, by excess adipose tissue-derived signaling molecules, including neprilysin and aldosterone (40). Obesity is more prevalent in females than in males (11.5% vs. 6.9%) (41), and its association with HF risk is greater in females, with an increased predilection for heart failure with preserved ejection fraction compared to heart failure with reduced ejection fraction (8, 42). Recent data highlight the importance of central obesity over BMI alone in adversely affecting the overall cardiac function, particularly in females (43, 44). Tobacco use is less prevalent in females than in males, with differences in influencing factors, including cultural, behavioral, and physiological responses. However, the independent risk association of smoking with HF among females has been shown to be almost double that among males (88% vs. 45%), based on the National Health and Nutrition Examination Survey data (45).

This study has some limitations. First, because this was a cross-sectional study, causal relationships could not be determined. Second, despite adjusting for several covariates, the potential impact of unmeasured or residual confounding factors may persist. Third, our data on both HF status and carotenoid intake were based on self-reports from participants, which may be subject to information bias.

Above all, increased efforts devoted to the promotion of awareness and widespread dissemination of known sex-based differences in HF are needed, with a focus on the gaps in knowledge that remain to be filled.

## 5 Conclusion

We found that in a sample of adults in the US, higher carotenoid intake was associated with a lower risk of HF in females, however, not in males. In addition, the sex-based differences were statistically significant. These findings warrant further validation through prospective studies, and additional research is needed to explore the mechanisms underlying these associations. Ultimately, these findings may help reduce the risk of HF and improve disease control by changing lifestyle and dietary factors.

## Data Availability

The datasets presented in this study can be found in online repositories. The names of the repository/repositories and accession number(s) can be found below: https://www.cdc.gov/nchs/nhanes/index.htm.
